# Cardioprotection Resulting from Glucagon-Like Peptide-1 Administration Involves Shifting Metabolic Substrate Utilization to Increase Energy Efficiency in the Rat Heart

**DOI:** 10.1371/journal.pone.0130894

**Published:** 2015-06-22

**Authors:** Karpagam Aravindhan, Weike Bao, Mark R. Harpel, Robert N. Willette, John J. Lepore, Beat M. Jucker

**Affiliations:** Heart Failure Discovery Performance Unit, Metabolic Pathways and Cardiovascular Therapeutic Area, GlaxoSmithKline, King of Prussia, Pennsylvania, United States of America; Thomas Jefferson University, UNITED STATES

## Abstract

Previous studies have shown that glucagon-like peptide-1 (GLP-1) provides cardiovascular benefits independent of its role on peripheral glycemic control. However, the precise mechanism(s) by which GLP-1 treatment renders cardioprotection during myocardial ischemia remain unresolved. Here we examined the role for GLP-1 treatment on glucose and fatty acid metabolism in normal and ischemic rat hearts following a 30 min ischemia and 24 h reperfusion injury, and in isolated cardiomyocytes (CM). Relative carbohydrate and fat oxidation levels were measured in both normal and ischemic hearts using a 1-^13^C glucose clamp coupled with NMR-based isotopomer analysis, as well as in adult rat CMs by monitoring pH and O_2_ consumption in the presence of glucose or palmitate. In normal heart, GLP-1 increased glucose uptake (↑64%, p<0.05) without affecting glycogen levels. In ischemic hearts, GLP-1 induced metabolic substrate switching by increasing the ratio of carbohydrate versus fat oxidation (↑14%, p<0.01) in the LV area not at risk, without affecting cAMP levels. Interestingly, no substrate switching occurred in the LV area at risk, despite an increase in cAMP (↑106%, p<0.05) and lactate (↑121%, p<0.01) levels. Furthermore, in isolated CMs GLP-1 treatment increased glucose utilization (↑14%, p<0.05) and decreased fatty acid oxidation (↓15%, p<0.05) consistent with *in vivo* finding. Our results show that this benefit may derive from distinct and complementary roles of GLP-1 treatment on metabolism in myocardial sub-regions in response to this injury. In particular, a switch to anaerobic glycolysis in the ischemic area provides a compensatory substrate switch to overcome the energetic deficit in this region in the face of reduced tissue oxygenation, whereas a switch to more energetically favorable carbohydrate oxidation in more highly oxygenated remote regions supports maintaining cardiac contractility in a complementary manner.

## Introduction

Glucagon-like peptide-1 (GLP-1) is an incretin hormone secreted by intestinal L-cells in response to nutrient ingestion [1]. GLP-1 regulates glucose homeostasis by stimulating insulin secretion, inhibiting glucagon secretion, delaying gastric emptying and promoting satiety [2]. Although the major physiological function of GLP-1 is associated with glycemic control, GLP-1 has further been shown to have beneficial impact on cardiovascular function independent of its effects on systemic glucose homeostasis [1,2]. Specifically, intravenous infusion of GLP-1 improved regional and global left ventricular (LV) function in patients with severe systolic dysfunction [3,4], and perioperative use of GLP-1 during coronary artery bypass grafting improved not only glycemic control but also hemodynamic recovery without requiring a high dose of inotropes [5]. In animals, GLP-1 infusion dramatically improved LV function and systemic hemodynamics in dogs with advanced dilated cardiomyopathy and enhanced recovery from ischemic myocardial stunning [6,7]. GLP-1 also reduced infarct size in isolated perfused rat hearts subjected to permanent coronary artery occlusion [8]. In addition, we and others have demonstrated that long acting GLP-1 agonists provide infarct-limiting effects following myocardial ischemia/reperfusion injury *in vivo* [9,10,11,12].

Several studies in preclinical animal models established that the cardiovascular benefits of GLP-1 are independent of its role in peripheral glycemic control [1,8,13–16]. Energy is mainly derived in the normal heart from cellular uptake of fatty acids and glucose and the subsequent metabolism of these substrates via beta-oxidation and aerobic/non-aerobic glycolysis, respectively [17]. The ability of the heart to switch from less energetically efficient fatty acid to more energetically efficient carbohydrate could be a useful predictor for protection in the failing heart. Indeed, energy-sparing treatments for the ischemic heart disease and heart failure such as beta receptor blockers, ACE inhibitors or angiotensin II receptor blockers have been shown to improve the disease prognosis [17]. Accordingly, GLP-1 has been shown to increase myocardial glucose uptake through an Akt-1-independent mechanism that is distinct from the actions of insulin [18]. However, the extent to which intermediary substrate metabolism plays a role in this cardioprotection, in particular whether GLP-1 modulates metabolic substrate shifting in different regions (i.e. ischemic versus remote areas) of the myocardium in response to injury has not been resolved. GLP-1 also has been shown to activate survival and intracellular signaling pathways that involve protein kinase B, ERK1/2 and AMP kinase in cellular [19] and in animal [20,21] models. However, it is not known whether this is a direct or indirect consequence of GLP-1 activation in the cardiomyocyte.

The objective of the present study was to investigate the mechanism(s) for cardioprotection following GLP-1 treatment from the perspectives of available metabolic substrate reserve and fuel switching in both area at risk (AAR) and area not at risk (ANAR) regions of heart following ischemic insult. This information is relevant for clinical investigation of potential GLP-1 based therapeutic strategies for diabetic cardiomyopathy and other myocardial diseases in which ischemic damage drives progressive injury and eventually heart failure. We provide evidence that an energetically-favorable metabolic substrate switch from use of predominantly fat oxidation to use of predominantly glucose and other carbohydrates occur in the ANAR of ischemia-reperfusion (I/R) injured rat hearts following treatment with GLP-1 peptide. Interestingly, this switch is matched to the availability of oxygen in these regions, with anaerobic glycolysis predominating in the AAR and carbohydrate oxidation in the more highly oxygenated ANAR. These findings are supported by cellular studies using cardiomyocytes (CMs) harvested from adult rats.

## Materials and Method

Male Sprague-Dawley rats (250-350g) from Charles River laboratory (Wilmington, DE) were used for the study. All experiments were conducted in accordance with protocols and guidelines approved by the Institutional Animal Care and Use Committee of GlaxoSmithKline. For in vivo studies, GLP-1 (7–36) (Phoenix Pharmaceuticals Inc, Cat# 028–11) was administered at a dose of 300 pmol/kg/min, and saline was used as the vehicle for GLP-1.

### Myocardial I/R injury

GLP-1 or vehicle was administered by IV infusion ten minutes prior to the induction of ischemia, followed by continuous subcutaneous infusion using an osmotic pump (Durect Corporation, Model-2ML Alzet pump) until the end of the reperfusion period.

Rats underwent 30 min myocardial ischemia followed by 24 hr reperfusion as reported previously [22,23]. Briefly, after anesthesia with Nembutal (60 mg/kg, intraperitoneal injection), rats were intubated with polyethylene-190 tubing and ventilated with a small-animal volume-controlled respirator at a tidal volume of 10 ml/kg and 90 cycles/min (Harvard Apparatus, Holliston, MA). The pain reflex was examined by pinching the tail or toe to indicate an appropriate anesthesia before skin incision. Artificial tear ointment was applied to the rat prior to the surgery to prevent corneal drying. For each animal, the chest was opened through a midline sternotomy and the heart was exposed using a small rodent retractor. A 7-0 suture was passed under the left anterior descending coronary artery (LAD) 1 mm below the left atrium to tie a non-traumatic balloon occluder for occlusion and reperfusion of the LAD. After 30 minutes of occlusion, the LAD artery was reopened by loosening the suture. However, the suture was not removed and used as a landmark to separate the area at risk (anterior LV wall below the suture) from the area not at risk (lateral and septal LV wall) when the heart was harvested. Successful coronary occlusion and reperfusion was verified by the color change in the apex and by observing S-T segment elevation and widening of the QRS on the ECG during ischemia and resolution after reperfusion. The incision was closed by layers using 5-0 suture. The endotracheal tube was removed after spontaneous breathing recovered. Post-surgery, animals were given saline ip to replace the loss of fluid, provided nasal oxygen, and kept warm in a 37°C incubator until fully ambulatory, at which point they were returned to their home cages in the animal facility.

### Hemodynamics and infarct size

At the end of the I/R study (24 hr post-reperfusion), animals were anesthetized with isoflurane (2% in oxygen) and a 2 F Millar Mikro-tip catheter transducer was inserted into the left ventricle (LV) through the right carotid artery to measure LV pressure and the derived maximum rate of rise and fall in left ventricle pressure (+dP/dt_max_ and-dP/dt_min_, respectively), as described previously [22,23]. After hemodynamic measurements, the heart was excised and perfused with saline and then a 1% solution of 2,3,4-triphenyltetrazolium chloride (TTC) in phosphate buffer (pH 7.4, 37 ⁰C). The viable myocardium stained red while the infarcted myocardium was white. The AAR and ANAR were identified by next perfusing 1% Evans blue dye in normal saline. The infarcted, AAR and ANAR regions were traced and measured using Image-Pro Plus software. Infarct size was presented as percentage of the AAR and AAR was presented as percentage of left ventricle.

### *In vivo* glucose uptake

Glucose uptake was determined using the [^3^H]-2-deoxyglucose (2DG) method described with slight modification [24]. Pre-cannulated rats purchased from Charles River laboratory were anesthetized with isoflurane (4%) following an overnight fast. After an initial blood draw for baseline glucose measurements, rats were administered saline, insulin (3 U/kg) or GLP-1, followed by a tracer bolus dose of 2DG at 100 μCi/kg. In animals receiving insulin, glucose was infused at 14 mg/kg/min from 10 min prior to the 2DG challenge until the end of the 30-min uptake period to prevent hypoglycemia. In animals receiving GLP-1 peptide, the peptide infusion was initiated 5 min prior to 2DG administration; no exogenous glucose was administrated as GLP-1 did not produce any hypoglycemic response.

Plasma samples were collected every 5 minutes through the infusion period, deproteinated and quantified for radioactive content by diluting 200 μl of each sample with 1 ml of Optiphase supermix (Perkin Elmer, Waltham, MA) and measured using scintillation counting with a Microbeta Trilux detector (Perkin Elmer model 1450-02). Raw counts are reported. Hearts were freeze clamped at the end of the infusion period, extracted with perchloric acid and then similarly subjected to scintillation analysis for radioactive content. Tissue [^3^H]-2DG-6-P (“trapped” intracellular form of 2DG) was calculated as the difference between tissue radioactivity (representing “total” 2DG *plus* 2DG-6-P resulting from 2DG uptake and subsequent phosphorylation) and plasma radioactivity (representing “free” 2DG equivalent to plasma volume in the tissue). Glucose disposal flux was determined using the average plasma glucose concentration, terminal heart tissue radioactivity, and the area under the curve for plasma radioactivity as previously described [25]**.**

### Cardiac metabolic flux

Relative carbohydrate versus free fatty acid (FFA) oxidation was assessed following an *in vivo* glucose clamp experiment in which rats were administered IV vehicle or GLP-1 10 min prior to I/R injury or simultaneously with the initiation of euinsulinemic-hyperglycemic clamp in control, non-injured animals. For the glucose clamp experiment, rats (n = 5-6 per group) were subjected to a euinsulinemic-hyperglycemic clamp (continuous [1-^13^C] glucose (8 mg/kg/min)/somatostatin (1.5 μg/min) infusion via jugular vein) lasting for 120 minutes. The glucose infusion rate approximately matched the hepatic glucose production rate and insulin was clamped at 1072±82 pg/ml resulting in a plasma glucose concentration of 260–300 mg/dL. This period was sufficient for glycolytic and tricarboxylic cycle intermediates to achieve steady-state enrichments. At the end of the clamp experiment, the hearts were rapidly removed and LV separated into AAR and ANAR regions before placing in liquid N_2_. Perchloric acid extracts were prepared from frozen heart sections, neutralized with KOH, lyophilized, and subsequently dissolved in 500 μl D_2_O. Proton Observe Carbon Enhanced (POCE) ^1^H MRS measurements of metabolite ^13^C enrichments in tissue extracts were performed at 9.4T spectrometer as previously reported [23,26,27]. Relative cardiac carbohydrate (including glucose, glycogen, pyruvate, and lactate) and fat (FFA and ketone) oxidations in terms of relative substrate contribution to acetyl-CoA oxidation were assessed from the metabolite pool enrichments: Relative carbohydrate oxidation rate = (4-^13^C glutamate enrichment)/((3-^13^C lactate + 3-^13^C alanine)/2; relative fat oxidation = 1- (4-^13^C glutamate enrichment)/((3-^13^C lactate + 3-^13^C alanine)/2.

### Langendorff perfused isolated heart

The perfusion protocol was performed as described earlier [28]. After a period of stabilization with Krebs Henseleit (KH) buffer, baseline buffer solution was collected to determine the initial glucose and lactate concentrations prior to perfusing the heart. The effluent was collected over 1 min, every 10 min during a 30 min treatment period and the coronary flow was calculated by a timed collection of the effluent. At the end of the 30 min perfusion period with GLP-1 (500 pM) or insulin (100 U/L) the effluents were collected for glucose and lactate analysis. The glucose and lactate flux was determined as previously described in [18,28] and flux was reported as mg/g wet weight/30 min.

### cAMP, glycogen, glucose, lactate and active GLP-1 levels

For cardiac analytes, heart tissue samples were pulverized in liquid nitrogen and saved in -80 ⁰C degree freezer until analysis. cAMP in tissue was extracted using 5% trichloroacetic acid and ether as described previously [29] and measured using a competitive enzyme immunoassay from Cayman Chemical (Cat#581001, Ann Arbor, MI).

Plasma and tissue glucose and lactate concentrations were analyzed using an Olympus AU640 analyzer (Olympus America Inc., Melville, NY) using reagents from Olympus. Cardiac glycogen was measured in concentration of glucosyl units as previously described [30]. Active GLP-1 in plasma was measured using a GLP-1 EIA kit (Linco Research Inc, Cat#EGLP-35K) in the presence of (Dipeptidyl Peptidase-4) DPP4 (EMD Millipore Corporation, Cat#DPP4) in either diluted or undiluted plasma samples from the *in vivo* studies.

### Cell culture

CMs were isolated from the hearts of adult rats (300–350 g) using collagenase-based enzymatic digestion in a slightly modified method described previously [31]. In brief, hearts were excised and initially perfused for 5 min with Krebs buffer (solution-A) containing 130 mM NaCl, 4.5 mM KCl, 0.4 mM NaH_2_PO_4,_ 1.4 mM MgCl_2_, 4.2 mM HEPES, 20 mM taurine, 10 mM creatine and 10 mM glucose. The initial perfusion was followed by enzymatic digestion in solution-A containing 100 μM CaCl_2_ and 1 mg/ml collagenase (Worthington type II) for 30 min at 37°C. The ventricles were cut into small pieces and myocytes were separated from undigested tissue by filtering through nylon gauze. The myocytes were allowed to settle into a pellet in solution-A in the presence of 2% BSA. The myocytes were readapted to nominal calcium by allowing them to settle into a pellet at room temperature in solution-A containing 0.2 mM, 0.4 mM, 0.8 mM and 1 mM sequentially. CMs were seeded for various experiments on laminin coated plates in Dulbecco’s modified Eagle’s medium, pH 7.4 (DMEM) with glucose (5 mM) pyruvate (1 mM), L-Glutamine (4 mM), taurine (5 mM), creatine (5 mM), carnatine (2 mM) and 0.2% BSA, optimal maintenance medium or in DMEM with glucose (25 mM), L-Glutamine (2 mM), taurine (5 mM), creatine (5 mM), carnatine (2 mM) and 0.2% BSA, suboptimal medium and used within 24 hr in culture. During the bioenergetics measurement cells in unbuffered DMEM medium were either given insulin (70 nM) or GLP-1 (up to 100 nM) through the XF24 Analyzer injection ports.

### Bioenergetics measurement

An XF24 Analyzer (Seahorse Biosciences) was used to measure the bioenergetic profile of intact CMs in terms of real-time oxygen consumption rate (OCR), extracellular acidification rate (ECAR) and proton production rate (PPR).

For all bioenergetic measurements the CMs were seeded at 8K one day prior to the experiment, and incubated for one hour in serum free unbuffered DMEM in a non-CO_2_ incubator as per the manufacturer’s instructions. These seeding densities were optimized to provide a baseline OCR around 200–300 pmol/min. For glucose utilization measurements, the unbuffered DMEM medium supplemented with L-glutamine (2 or 4 mM), with or without sodium pyruvate (1 mM) and glucose (5 mM) was used. Change in ECAR following treatment with either Insulin or GLP-1 was recorded as a measure of glycolysis and glucose oxidation. For fatty acid oxidation measurements, KHB medium containing 111 mM NaCl, 4.7 mM KCl, 2 mM MgSO4, 1.2 mM Na_2_HPO_4_ and supplemented with 2.5 mM glucose and 0.5 mM carnitine. Changes in OCR were recorded following palmitate (50 μM) conjugated to BSA (6:1 molar ratio) addition as a measure of fatty acid oxidation (Ferrick et al., 2008). Glycolysis and fatty acid oxidation assays were validated using ATP synthase inhibitor (oligomycin) and carnitine palmitoyltransferase-1 inhibitor (etomoxir) respectively. Mitochondrial integrity was tested using respiratory complex inhibitors oligomycin, carbonyl cyanide 4-(trifluoromethoxy) phenylhydrazone (FCCP), and rotenone and monitoring OCR changes to assess ATP linked oxygen consumption, proton leak, reserve capacity and non mitochondrial respiration as described previously [32].

### Statistical analysis

All data are presented as mean±SEM. All statistical tests were performed using GraphPad Prism 6. An unpaired Student’s t-test or one-way ANOVA with Newman-Keuls *post hoc* test was used to evaluate the level of significant difference between multiple groups. p<0.05 was considered significant.

## Results

### *In* vivo cardioprotection following I/R injury

GLP-1 was tested in the rat I/R model in order to study its cardioprotective effects in the presence of optimal insulin stimulation [33]. The plasma GLP-1 concentration at 30 min post-GLP-1 infusion increased to 212±2 pM from 5±1 pM at baseline.

**Fig 1:**
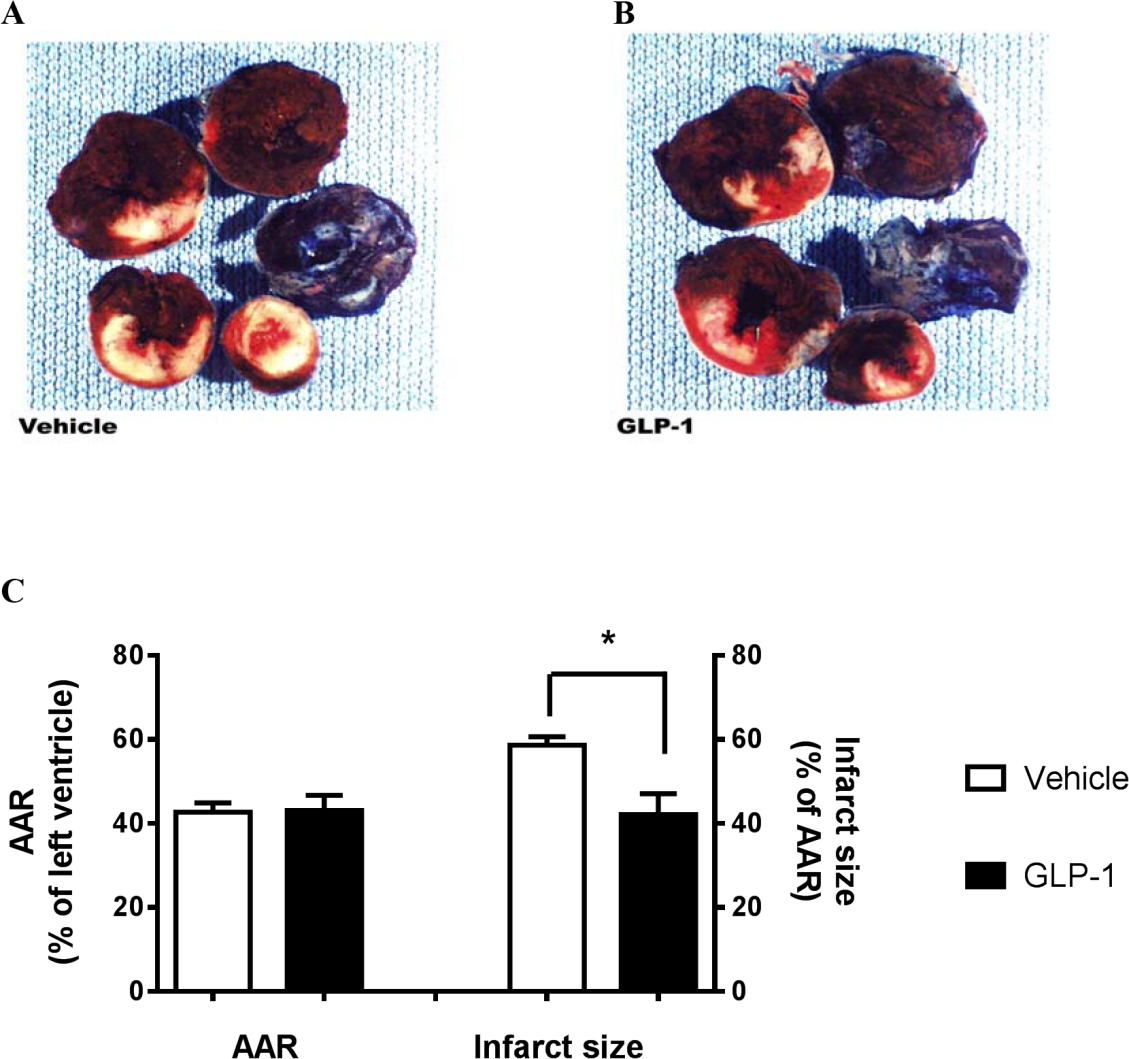
Infarct assessment following cardiac ischemia-reperfusion injury in rat. Sprague-Dawley rats (n = 5-6) were subjected to a 30 min LAD coronary artery occlusion followed by 24 hr period of reperfusion. Hearts were harvested for assessment of area at risk and infarct size. Representative photographs of heart sections stained with TTC and Evans Blue dye are shown for both Vehicle (A) and GLP-1 (B) groups. The areas of myocardial infarct are white, areas at risk (AAR) are the combined white and red regions, and area not at risk (ANAR) are dark blue. Infarct size and area at risk are presented as percentage of AAR and left ventricle, respectively (C). Data are presented as mean±SEM. *p<0.05 vs Vehicle.

Differential staining of LV with TTC and Evans Blue dyes was used at the end of the reperfusion period to delineate the affected areas and determine the extent of myocardial damage. Representative photographs of heart sections are shown in (Fig 1A and 1B)**.** The area of myocardial infarction appears white; AAR, white and red; ANAR, dark blue. As quantified in (Fig 1C), AAR was similar between vehicle and GLP-1 treatment groups, suggesting that both groups underwent similar ischemic insults. The infarct size in the vehicle group of 58.6±2.0% of AAR was reduced significantly with GLP-1 treatment to 42.1±5.0% of AAR.

There was no difference in heart rate, LVEDP or LVSP between the vehicle and GLP-1 treated animals. However, the contractile function presented as +dP/dt_max_ was significantly enhanced with GLP-1 treatment compared to vehicle (Table 1).

**Table 1:** Left ventricle hemodynamics following I/R injury.

	Vehicle	GLP-1
	(n = 6)	(n = 6)
LVSP (mmHg)	90±5.4	105±2.4
LVEDP (mmHg)	10.2±0.9	9.5±0.85
HR (bpm)	386±18	391±8
+dP/dt_max_	5870±333	6982±179*
-dP/dt_min_	4594±344	5532±29

All data are presented as mean±SEM. LVSP, left ventricle systolic pressure; LVEDP, left ventricle end diastolic pressure; HR, heart rate; +dP/dt_max_, maximum rate of left ventricle pressure rise;-dP/dt_min_, maximum rate of left ventricle pressure fall.

*p<0.05 vs Vehicle.

**Fig 2:**
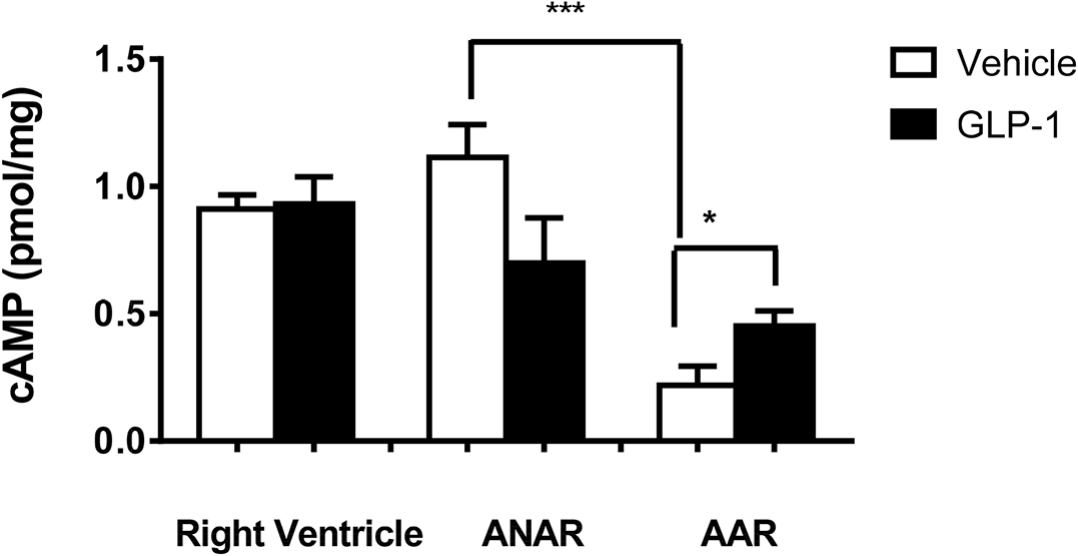
Tissue cAMP levels in the right ventricle, AAR, and ANAR of left ventricle after myocardial ischemia/reperfusion injury. Tissues were harvested following a 30 min ischemia/24 h reperfusion period and extracted as described in the text for cAMP analysis. Data comparing Vehicle (n = 3-6) with GLP-1 (300 pmol/kg/min) (n = 3-6) treatment groups are shown, with mean ±SEM as indicated. ***p<0.001 vs ANAR *p<0.05 vs Vehicle.

Cardiac cAMP levels were determined in left and right ventricles in order to probe the effects of GLP-1 treatment in remote versus ischemic areas of the heart. As shown in (Fig 2), cAMP remained unchanged in right ventricle between the vehicle treatment (0.91±0.06 pmol/mg) and GLP-1 treatment (0.93±0.11 pmol/mg) groups. The level of cAMP was slightly, but not significantly lower in the ANAR of the GLP-1 treated group (0.70±0.18 pmol/mg) compared to vehicle (1.12±0.13 pmol/mg). In contrast, cAMP increased from 0.22±0.07 to 0.45±0.06 pmol/mg of wet weight in the AAR with GLP-1 treatment (p<0.05).

### Cardiac metabolic profiling

**Fig 3:**
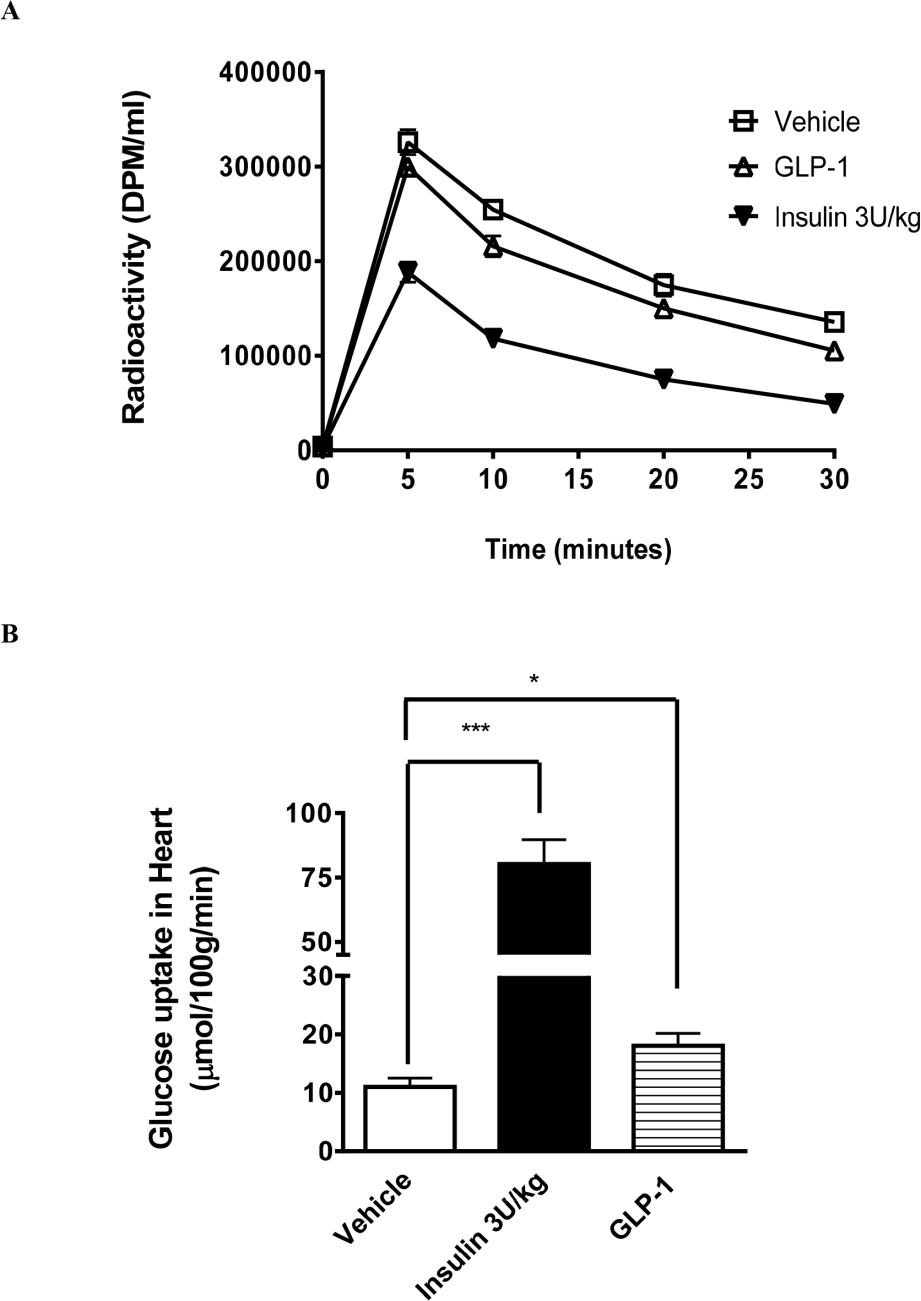
*In vivo* [^3^H]-2-DG uptake in rat myocardium. Kinetics of [^**3**^H]-2-DG clearance from plasma after a single bolus injection for Vehicle (, n = 8), GLP-1 (300 pmol/kg/min) (∆, n = 8), or insulin (3 U/kg) (▼, N = 5) (A). Myocardial glucose uptake measured at the end of study; i.e. 30 min following tracer administration (B). Data are presented as mean±SEM. *p<0.05, ***p<0.001 vs Vehicle.

Glucose disposal in the uninjured heart was assessed *in vivo* by monitoring 2DG clearance from blood and measuring uptake into cardiac tissue in rats receiving a continuous infusion of vehicle, GLP-1 or insulin (3 U/kg) comparator. A slightly accelerated rate of 2DG (*i*.*e*. glucose) clearance from blood with GLP-1 treatment versus vehicle (Fig 3A) translated to a significant increase in myocardial glucose uptake rate (from 10.5±1.7 to 18.0±2.1 μmol/100 g/min; (p<0.05) (Fig 3B). Insulin elicited an even higher rate of both plasma clearance and tissue uptake of 2DG (80.3±9.4 μmol/100 g/min; p<0.001).

The effects of GLP-1 treatment on cardiac glucose disposal were also evaluated by monitoring glucose and lactate flux in a Langendorff perfused heart preparation using continuous perfusion of GLP-1 at 500 pM (the maximum effective dose used in earlier reports [18,33], vehicle or insulin (100 U/L). Myocardial glucose uptake increased from 3.8±0.7 to 7.0±1.4 mg/g/30 min in vehicle and GLP-1 treated animals (84% increase; p<0.05), and lactate production increased from 1.3±0.4 to 3.5±0.8 mg/g/30 min in vehicle and GLP-1 treated animals respectively (169% increase; p<0.05). The effect observed with GLP-1 was similar to that observed with insulin (e.g. increases in glucose uptake and lactate production to 12.2±0.2 and 5.4±0.9 mg/g/30 min, respectively; p<0.01).

**Fig 4:**
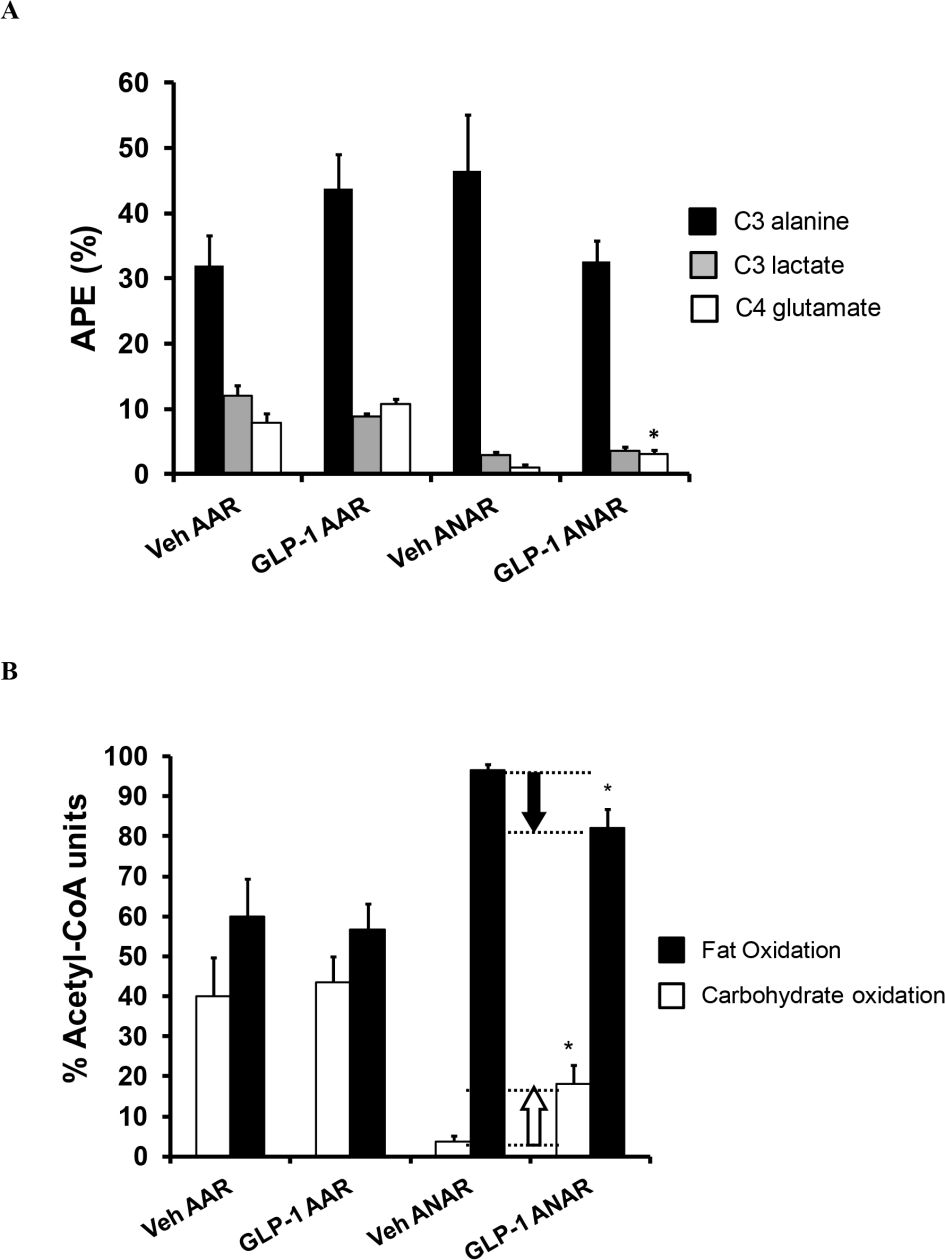
*In vivo* intermediary glucose metabolism in AAR and ANAR of rat left ventricle following a euinsulinemic-hyperglycemic clamp over 2 hr with 1-[^13^C] glucose infusion. Left ventricle intermediary metabolite ^**13**^C enrichments of alanine, lactate and glutamate are presented for AAR or ANAR myocardial tissue from Vehicle (n = 6) and GLP-1 (300 pmol/kg/min group, n = 6) treatment groups (A). Relative carbohydrate oxidation versus fat oxidation in Vehicle and GLP-1 treated AAR and ANAR myocardial tissue (B). The relative carbohydrate versus fat oxidation is calculated using isotopomer analysis of alanine and glutamate enrichments as described in the methods section. Data are presented as mean±SEM. *p<0.05 vs respective Vehicle.

The effects of GLP-1 treatment on metabolic substrate oxidation within AAR and ANAR regions of the left ventricle following cardiac I/R injury were determined by [1-^13^C] glucose euinsulinemic-hyperglycemic clamp. A significant increase in [4-^13^C] glutamate enrichment (2.9 fold, p<0.05) was observed in the ANAR region, without changes in AAR, following GLP-1 treatment (Fig 4A). There were no differences in either [3-^13^C] lactate or [3-^13^C] alanine enrichment in the two regions following treatment with GLP-1. Isotopomer analysis of alanine, lactate and glutamate enrichments indicated that treatment with GLP-1 resulted in a significant increase in relative carbohydrate oxidation versus fat oxidation in the ANAR region (p<0.05, Fig 4B); carbohydrate oxidation (e.g. glucose, glycogen, and lactate oxidation) increased from 3.5% to 18.0% and fat oxidation decreased from 96.5% to 82.0%. However, GLP-1 treatment had no effect on altering the metabolic profile in the AAR region of the myocardium. It should be noted that the ^13^C enrichment (APE) of a metabolite pool (e.g. lactate, alanine, glutamate) simply reflects the amount of ^13^C label in that metabolite pool, but does not reflect actual metabolite concentration. In addition, metabolic flux as measured by Langendorff perfused heart or metabolite labeling experiments can be altered independently of metabolite concentration changes. Therefore, there can be an increase in glucose disposal and/or lactate production and release from the heart while steady state intracellular glucose or lactate concentrations may not increase or not increase substantially.

The cardiac lactate, free glucose and glycogen pools were measured in the hearts of uninjured rats treated for 2 hours with GLP-1 or cardiac I/R injured rats treated with vehicle or GLP-1 through the entire duration of the I/R insult. There were no significant changes in cardiac lactate, free glucose or glycogen levels between GLP-1 treatment and vehicle in the control hearts (Table 2). These data suggest that while glucose uptake and oxidation in normal or ANAR cardiac tissue increases upon GLP-1 stimulation, intracellular concentrations of glucose do not have to increase or increase substantially. In fact, hexokinase is tightly coupled with glucose in the heart such that little free glucose exists in normal heart even when there is increased transport into the heart [34]. However, free glucose increased significantly by 1.5-fold (p<0.01) and lactate decreased significantly by 2.4-fold (p<0.001) in the AAR region compared to ANAR region of the left ventricle in vehicle-treated hearts from I/R injured animals, possibly reflecting decreased anaerobic glycolysis in the AAR region. The cardiac lactate concentration in the AAR region was normalized to that of ANAR region following treatment with GLP-1, possibly reflecting an increase in anaerobic glycolysis in the ischemic region of these hearts.

**Table 2:** Cardiac free glucose, lactate and glycogen in normal or AAR and ANAR regions of I/R injured hearts.

		Vehicle	GLP-1
		(n = 5–6)	(n = 5–6)
Free glucose (μmol/g)	Normal	2.5±0.2	2.0±0.4
	ANAR	3.3±0.4	2.7±0.1
	AAR	5.0±0.4^++^	4.1±0.2* ^+^
Lactate (μmol/g)	Normal	14.1±1.2	12.4±1.8
	ANAR	11.9±0.8	11.7±0.6
	AAR	5.0±1.0^+++^	11.1±1.3***
Glycogen (μmol/g)	Normal	11.4±3.5	12.6±1.6

All data are presented as mean±SEM. AAR, area at risk; ANAR, area not at risk.

+p<0.05

++p<0.01

+++p<0.001 vs ANAR

*p<0.05

***p<0.001 vs Vehicle.

### Cellular bioenergetics

**Fig 5:**
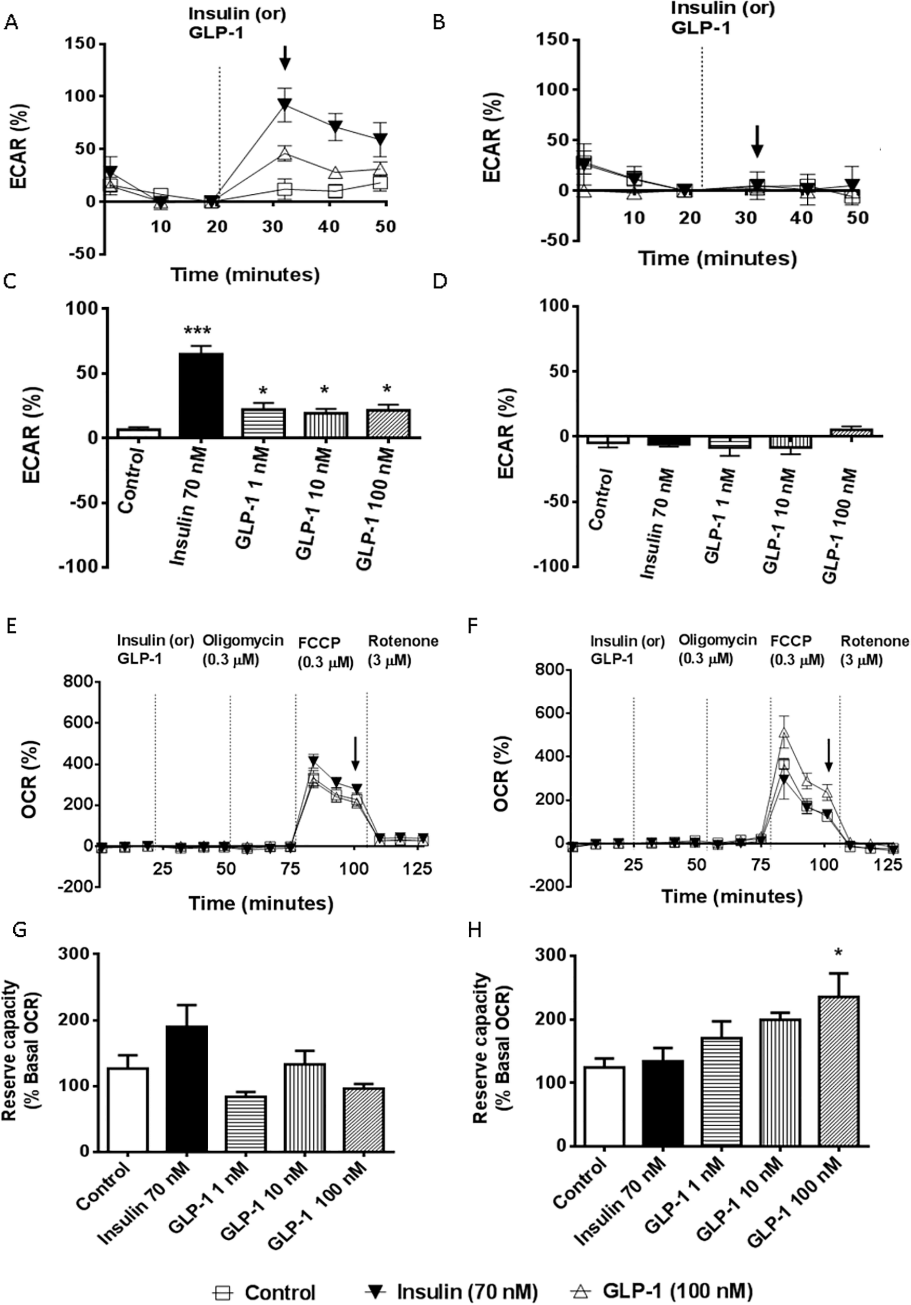
Glucose utilization and reserve capacity in cultured CMs. Glucose utilization was assessed by examining percent change in ECAR and the reserve capacity assessed as percent change in OCR following FCCP challenge in the presence of indicated concentrations of GLP-1 or insulin at 70 nM. A typical seahorse plot representing changes in ECAR over time following acute treatment with 100 nM GLP-1 (maximal effective dose) or insulin optimal media (A) or suboptimal media (B). Percentage change in ECAR, 10 min post injection of GLP-1 (1, 10, 100 nM) or insulin with optimal media (C) and suboptimal media (D). Typical seahorse plots representing changes in OCR over time following acute treatment with 100 nM GLP-1 or insulin with optimal (E) or suboptimal (F) media. Percentage change in OCR, 80 min post injection of GLP-1 (1, 10, 100 nM) or insulin in optimal (G) and suboptimal (H) media. ECAR, extracellular acidification rate; OCR, oxygen consumption rate. Data are presented as mean±SEM of 3–5 replicates per treatment from 2–4 individual experiments. ***p<0.001, *p<0.05, vs Control.

The observed substrate switching in ANAR following GLP-1 treatment was further investigated in CMs using an extracellular flux analyzer to measure the oxidation of glucose and fatty acid substrates. ECAR, as a measure of both lactic acid and CO_2_ formation during glycolysis and glucose oxidation respectively [35], was monitored in CMs cultured in optimal or suboptimal medium to make them either sensitive or insensitive to insulin respectively. In the optimal medium, GLP-1 at all concentrations tested from 1 nM to 100 nM increased ECAR by net 14%, (p<0.05, Fig 5A and 5C). This effect was significantly less than the 58% net increase observed with insulin (p<0.001) and consistent with the different levels of glucose uptake observed in the *in vivo* 2DG experiments. However, no significant change in ECAR was observed with either insulin or GLP-1 when tested in suboptimal medium (Fig 5B and 5D). CMs were also sequentially challenged with oligomycin, FCCP and rotenone to assess the effect of GLP-1 treatment on ATP turnover/proton leak, reserve capacity and non-mitochondrial respiration, respectively. A slight, but significant improvement in reserve capacity was observed with GLP-1 at 100 nM, but only in CMs maintained in suboptimal media lacking pyruvate (Fig 5F and 5H) and not when maintained in optimal media (Fig 5E and 5G). The reserve capacity or coupling efficiency improved from 124.4±14.2% in control to 235.5±32.0% of basal respiration in GLP-1 (100 nM) treated cells (p<0.05, Fig 5H). No significant change in ATP turnover, proton leak or non-mitochondrial respiration was observed.

**Fig 6:**
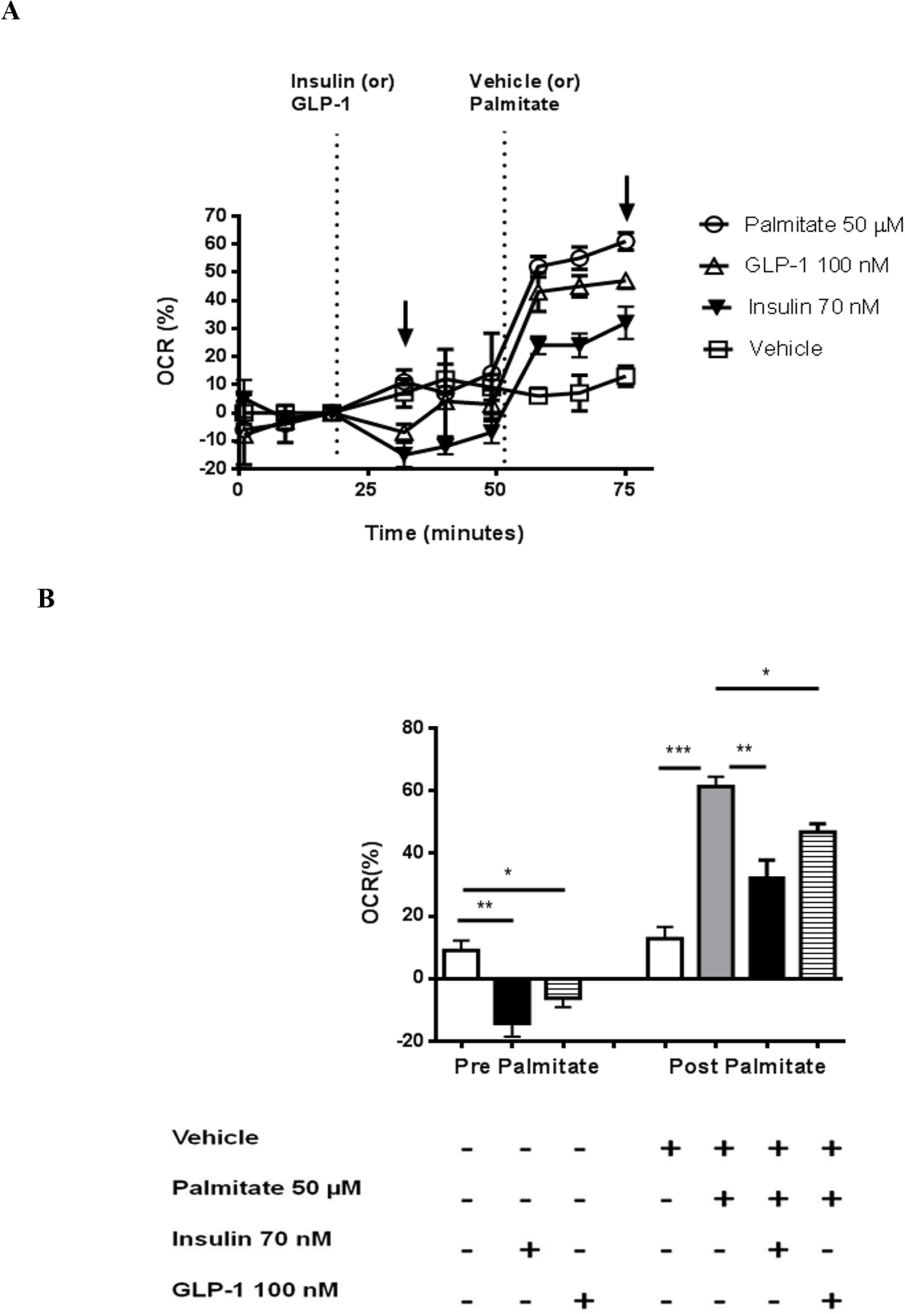
Effect of fatty acid oxidation on GLP-1 induced oxygen consumption in cultured CMs. OCR changes following stimulation with 100 nM GLP-1 (maximal effective dose) or 70 nM insulin are presented following palmitate challenge. GLP-1 or insulin was injected at 21 min and Vehicle (bovine serum albumin) or Palmitate was injected at 52 min following initiation of data collection. The responses are presented as percentage changes in OCR before and after Vehicle or Palmitate injection (A). Quantitation of percentage changes before (left bars; first arrow in panel A) and after (right bars; second arrow in Panel A) Palmitate or Vehicle challenge (B). Data are presented as mean ±SEM of 3–4 replicates per treatment. *p<0.05, **p<0.01, ***p<0.001.

The effects of GLP-1 treatment on fatty acid oxidation assay in CMs were observed as changes in OCR from baseline upon challenge with palmitate in the presence of low (2.5 mM) glucose. A small and transient decrease in OCR was observed upon addition of GLP-1 or insulin (by net 16% and 23% from control, respectively; Fig 6A and 6B); this transient response was lost within 10 min for GLP-1. Following challenge with palmitate, OCR decreased from 61.3±3.2% (vehicle) to 46.8±2.7% (p<0.05) and 32.3±5.6% in the presence of GLP-1 and insulin, respectively (Fig 6B), reflecting a decrease in fatty acid oxidation in each case.

## Discussion

The present study demonstrates a metabolic role for GLP-1 treatment resulting in cardioprotection on remote and ischemic rat myocardium following cardiac I/R injury. A shift to more energetically favorable carbohydrate oxidation in remote ANAR regions of the myocardium, and an increase in anaerobic glycolysis in the AAR regions may be important drivers for the observed improvement in cardiac function. A switch from fat to carbohydrate utilization *in vivo* was further confirmed in *in vitro* studies using CMs. The reserve capacity, a parameter of mitochondrial coupled respiration, increased slightly but significantly in GLP-1 treated CMs without affecting ATP turnover rate, and proton leak in these mitochondrial rich cells maintained in suboptimal medium. While this study was not designed to address whether the observed effects were GLP-1 receptor mediated, this study does confirm that GLP-1 treatment is capable of independently altering the metabolic and bioenergetics profile and contractility in ANAR and AAR regions of myocardium.

The degree of cardioprotection in the present study with GLP-1 corroborated results from previously published *in vivo* and *ex vivo* heart studies [1,8,13,36]. However, we now expand these findings by demonstrating that GLP-1 treatment shifts the fuel source towards increased carbohydrate oxidation while maintaining the predominant fat oxidation in the ANAR region of the myocardium, but not in the AAR region of the myocardium. While other investigators have previously shown that GLP-1 can directly stimulate glucose uptake [21] and oxidation [37] in the heart, the differential metabolic effects of GLP-1 in ischemic versus remote regions of the heart has not previously been described. This metabolic shift is critical for increasing energetic efficiency in the heart. Previous reports suggest no increase in glucose uptake and glycolysis with GLP-1 in isolated heart in the presence of oleic acid [38] or in adult rat ventricular myocytes [39]. In the current study, oleic acid was not used in the isolated perfused heart preparation in order to avoid inhibition of glycolysis. We used a crude CM preparation instead of percoll purified CM which are enriched for ventricular mycocytes. Further T3 was not used in the CM study since T3 appears to increase fatty acid oxidation in human induced pluripotent stem cell derived cardiomyocytes. Therefore the observations made in the current study may be due to the different model/condition used compared to that used in other labs. Consistent with the *in vivo* findings in the current study, GLP-1 treatment increased energy efficiency by decreasing oxygen consumption in the presence of stimulated fatty acid oxidation in CMs under normoxic conditions. While direct cardiac efficiency was not measured in vivo, contractility increased in the GLP-1 treated hearts with increased glucose uptake and relative carbohydrate oxidation. Therefore one can speculate that there was increased efficiency with GLP-1 treatment as glucose oxidation is ~12% more energetically efficient than fatty acid oxidation [40].

A recent report suggests that the insulin-like actions of GLP-1 on heart and vasculature may be mediated at the level of mitochondrial function such as by regulation of oxidative phosphorylation, ROS formation, gluconeogenesis, and fatty acid oxidation [41] and could have beneficial effects on the prevention or amelioration of lipotoxic cardiomyopathy [42]. We measured the bioenergetics of CMs following treatment with either GLP-1 or insulin in the presence of mitochondrial respiratory chain inhibitors: oligomycin, FCCP and rotenone. When CMs were maintained in media containing normal supplements with 1 mM sodium pyruvate, GLP-1 and insulin increased glycolysis/glucose oxidation and this response was found to be consistent with that observed in ANAR region of the myocardium with respect to increased carbohydrate oxidation. However no change in ATP turnover, proton leak, or reserve capacity was observed with either insulin or GLP-1 treatment up to 100 nM under optimal assay condition. When CMs were maintained in suboptimal media with no added pyruvate, glycolysis was not enhanced with either GLP-1 or insulin. This may be a result of glycolytic inhibition of pyruvate through the Randle mechanism [43] or could be due to oxidative stress since pyruvate is not only added to the medium as an energy supplement but also added to protect cells in culture from oxidative stress. Without added pyruvate, the CMs appeared insulin insensitive and mitochondrial respiration appeared less coupled, characteristics similar to that observed in AAR region of the myocardium. The respiratory reserve capacity or coupled respiration slightly improved when CMs were treated with GLP-1 in suboptimal medium tested. The lack of an effect with insulin under these conditions suggests that GLP-1 may be more efficient in terms of rescuing mitochondrial reserve capacity (coupled respiration) in insulin resistant myocardium. An *in vivo* model to verify such an effect in failing myocardium though useful might be difficult to setup since this requires efficient perfusion of GLP-1 where vascular integrity is compromised in cardiac injury. Further research is warranted to elucidate how GLP-1 treatment improves coupled respiration. Furthermore, cardiac lactate, free glucose and glycogen levels were unchanged in uninjured hearts during GLP-1 administration. However, injury-induced alterations in the free glucose and lactate pools in the AAR region of I/R-injured myocardium were significantly normalized following treatment with GLP-1, further associating the cardioprotective effect not only with increased carbohydrate oxidation in the ANAR region, but also with potentially increased anaerobic glycolysis as reflected by the increase in cardiac lactate observed in the AAR region of the heart. While GLP-1 treatment resulted in ~30% reduction in infarct size, the lactate concentration was increased by over 100% in the AAR region (5.0 vs 11.1 μmol/g in Vehicle vs GLP-1 respectively), so increased tissue viability may in part be responsible for the observed increase in AAR lactate.

A number of *in vitro* observations suggest that GLP-1 mediated cardioprotective effects in the cardiac I/R model are mediated by the activation of RISK pathway kinases such as PI3K, ERK1/2, cAMP-dependent PKA, Akt and P70S6K [44]. The RISK pathway inhibits MPTP opening, blocking calcium overload and upregulating several anti-apoptotic pathways. Our results show that downstream signaling following GLP-1 activation differs between the various regions of the injured LV. Compared to vehicle, GLP-1 normalized cAMP level in the AAR but slightly (although non significantly) decreased cAMP level in the ANAR. While the increase in cAMP may be a result of GLP-1 treatment effects in the AAR region to promote anaerobic glycolysis under high energy demand, this may also be an indirect consequence of having a more viable myocardium due to the decreased infarcted area which may be secondary to the increased anaerobic glycolysis observed in this region. These results presumably drive the negative inotropic response observed in normal rat myocardium [18]. The lack of cAMP response observed in ANAR region of the myocardium and in healthy CMs is consistent with what was reported earlier [21]. Our data together with that observed by others suggests differential signaling effects in healthy and injured myocardium.

In summary, our data show that the differential metabolic effects observed in ANAR and AAR regions of the ischemia injured heart may in large part contribute to the cardioprotection observed with GLP-1 treatment. While anaerobic glycolysis may contribute to ischemic cardioprotection, a shift to more energetically favorable carbohydrate oxidation in healthy, non-ischemic myocardium may play a beneficial role in maintaining cardiac contractility. These findings suggest that GLP-1 treatment results in a metabolic shift in viable regions of the heart which is critical for the preservation of myocardial function following ischemic injury.
